# Beyond evidence-based data: scientific rationale and tumor behavior to drive sequential and personalized therapeutic strategies for the treatment of metastatic renal cell carcinoma

**DOI:** 10.18632/oncotarget.7267

**Published:** 2016-02-08

**Authors:** Lorena Incorvaia, Giuseppe Bronte, Viviana Bazan, Giuseppe Badalamenti, Sergio Rizzo, Gianni Pantuso, Clara Natoli, Antonio Russo

**Affiliations:** ^1^ Department of Surgical, Oncological and Oral Sciences, Section of Medical Oncology, University of Palermo, Palermo, Italy; ^2^ Department of Surgical, Oncological and Oral Sciences, Section of Surgical Oncology, University of Palermo, Palermo, Italy; ^3^ Department of Medical, Oral and Biotechnological Sciences, University “G. D'Annunzio”, Chieti, Italy

**Keywords:** renal cell cancer, tyrosine kinase inhibitor, mTOR, angiogenesis, VEGFr

## Abstract

The recent advances in identification of the molecular mechanisms related to tumorigenesis and angiogenesis, along with the understanding of molecular alterations involved in renal cell carcinoma (RCC) pathogenesis, has allowed the development of several new drugs which have revolutionized the treatment of metastatic renal cell carcinoma (mRCC).

This process has resulted in clinically significant improvements in median overall survival and an increasing number of patients undergoes two or even three lines of therapy. Therefore, it is necessary a long-term perspective of the treatment: planning a sequential and personalized therapeutic strategy to improve clinical outcome, the potential to achieve long-term response, and to preserve quality of life (QOL), minimizing treatment-related toxicity and transforming mRCC into a chronically treatable condition.

Because of the challenges still encountered to draw an optimal therapeutic sequence, the main focus of this article will be to propose the optimal sequencing of existing, approved, oral targeted agents for the treatment of mRCC using evidence-based data along with the knowledge available on the tumor behavior and mechanisms of resistance to anti-angiogenic treatment to provide complementary information and to help the clinicians to maximize the effectiveness of targeted agents in the treatment of mRCC.

## INTRODUCTION

Renal cell carcinoma (RCC) has seen in last years a rapid and continuous increase of new cases [1]. Approximately a quarter of patients with RCC presents with locally advanced or metastatic disease at diagnosis, and about 20-40% of those with confined primary tumor will develop metastatic disease [2, 3].

The treatment of metastatic renal cell carcinoma (mRCC) has been revolutionized over the past 10 years, with the development of several new drugs, whose use has resulted in clinically significant improvements in median overall survival compared with the previous treatment options, limited to cytokines such as interleukin-2 and interferon α (IFN-α) [4]. These immunotherapies showed poor efficacy and severe dose-limiting toxicities [5, 6].

The recent advances in identification of the molecular mechanisms related to tumorigenesis, angiogenesis, cell growth and proliferation, along with the understanding of molecular alterations involved in RCC pathogenesis [7-12], allowed to identify targets of clinical interest: the vascular endothelial growth factor (VEGF) and its receptors (VEGFr), the mammalian target of rapamycin (mTOR) signaling pathway, the fibroblast growth factor (FGF) and its receptor (FGFr), the hypoxia-inducible factors (HIFs) and Akt activation.

**Figure 1:**
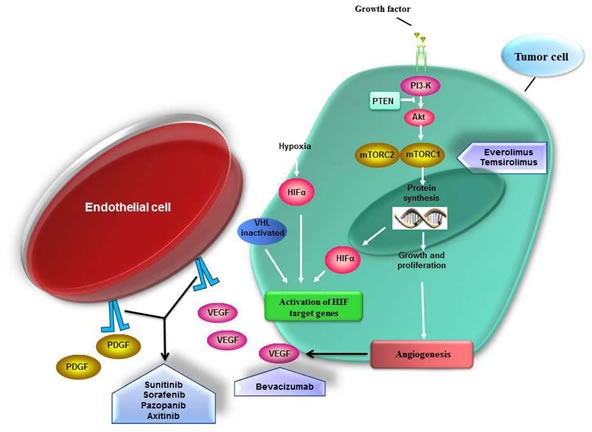
Signaling pathways inhibition by targeted agents in mRCC RCC is a highly vascularized tumor type. The chronic angiogenesis is required for growth of these tumors. Under normal conditions HIF (hypoxia inducible factor) is constitutively degraded. HIF promotes transcription of gene involved in the angiogenesis-pathway and tumor progression such as VEGF, PDGF and TGF-alfa. Inactivation of the von Hippel-Lindau (VHL) gene leads to accumulation of HIF transcription factors and subsequent activation of several mediator of angiogenesis. Also the activation of the mammalian target of rapamycin (mTOR) signaling pathway may result in HIF accumulation. Temsirolimus and everolimus are kinase inhibitors of mTOR complex 1 (mTORC1); bevacizumab is a humanized anti-VEGF monoclonal antibody; sorafenib, sunitinib, pazopanib and axitinib are multikinase inhibitor (TKIs) of growth factor receptors involved in the activation of angiogenesis-related pathways.

Based on these known signaling pathways, targeted systemic treatments have been specifically designed and approved: the humanized anti-VEGF monoclonal antibody bevacizumab [13, 14] in combination with IFN-α, four multitargeted tyrosine kinase inhibitors (TKIs): sorafenib, sunitinib, pazopanib and axitinib; and two kinase inhibitors of mTOR, temsirolimus and everolimus (Figure 1).

The introduction of these several new targeted agents raises many questions on how to use them in order to maximize their effectiveness and offer to the patient a greater number of therapeutic opportunities, in the most appropriate setting [15-18]: these are groups of agents with a different mechanism of action and consequently different toxicity, in the absence of molecular parameters able to predict response and resistance, and with few direct comparisons between them [19].

The data available often derived from studies planned to obtain drug registration, where different agents are compared with placebo or IFN-α, with little indirect differences among them in terms of progression-free survival (PFS) and overall survival (OS) [20].

Furthermore, although targeted agents have demonstrated PFS benefit, durable responses are rare and most patients with mRCC eventually experience disease progression [21, 22].

Therefore, in the new era of targeted therapies for the treatment of mRCC, it would be appropriate to plan a sequential and personalized therapeutic strategy to improve clinical outcome, to potentially achieve long-term response, and to preserve quality of life (QOL), minimizing treatment-related toxicity and transforming mRCC into a chronically treatable condition.

The main focus of this article will be to propose the optimal sequencing of existing, approved, oral targeted agents for the treatment of metastatic renal clear cell carcinoma, the most frequent subtype of sporadic RCC (70-85%) [23], although it remains an area of ongoing research, through the identification of novel pathways, new data from early clinical trials and emerging immune based therapies.

## EVIDENCE-BASED OUTLINE OF SEQUENCES OF TARGETED AGENTS

### First-line treatment

In the first-line treatment of mRCC population VEGF-targeted agents have improved patient outcomes compared with the previous cytokines-based standard of care.

Current evidence-based guidelines recommend three drugs as first-line treatment for patients with good-intermediate risk: bevacizumab (combined with IFN-α), sunitinib and pazopanib [3].

All three agents have demonstrated PFS prolongation, in phase III trials over either IFN-α or placebo [24-32].

First-line temsirolimus has demonstred activity in patients with poor prognosis [33].

Sunitinib, a multitarget oral TKI that inhibits the VEGF and PDGF receptors, was compared to IFN-α in a randomized trial of untreated patients, showing improved median PFS (11 vs 5 months) and median OS (26 vs 21 months) and a response rate over 40% [30]. These results allowed to set sunitinib as a standard treatment in this setting.

Pazopanib, another oral multitarget TKI with inhibitory activity against VEGF and PDGF receptor, was compared, head-to-head, with sunitinib, in a randomized non-inferiority trial in first-line setting (COMPARZ): the pazopanib and sunitinib groups had similar median PFS (8.4 months for pazopanib versus 9.5 months for sunitinib; HR, 1.047, 95% CI, 0.898-1.220) [34].

There were no significant differences in response rates and OS between the two agents (median overall survival 28.4 months in the pazopanib group and 29.3 months in the sunitinib group) [35].

The analyses of QOL were in favor of pazopanib.

The improved safety profile and QOL of pazopanib were confirmed by “The Preference Study of Pazopanib Versus Sunitinib in Advanced or Metastatic Kidney Cancer” (PISCES) which evaluated patients' preference for pazopanib or sunitinib in treatment-naive patients with mRCC: patients were randomized to pazopanib for 10 weeks, and after 2 weeks of washout, to sunitinib for 10 weeks, or to the opposite sequence.

At 22 weeks, patients completed a questionnaire assessing their preferences: significantly more patients preferred Pazopanib (70%) over Sunitinib (22%); 8% did not prefer any agent [36].

These findings have shown Sunitinib and Pazopanib are agents with similar efficacy in first-line treatment, but the use of Pazopanib as first line therapy, in patient with good-intermediate risk mRCC, seems more convenient for his better safety profile.

### Second-line treatment

Most patients initially treated with an anti-VEGF therapy in first-line setting, eventually develop resistance and subsequent disease progression. [37, 38]

Current post first-line therapies for mRCC, target the VEGFr and mTOR pathways. Approved treatment options include sorafenib, axitinib and everolimus. Sunitinib is also an option if it was not previously administered [3].

Sorafenib is a multikinase inhibitor of multiple growth factor receptors as VEGFr, PDGFr, Flt-3 and c-Kit and Raf-1, a member of RAF/MEK/ERK signaling pathway [39]; axitinib is a next-generation TKI, potent and highly selective for the VEGF receptor 1, 2 and 3 [40-42]; everolimus is a mammalian target of mTor inhibitor (mTORi) [43].

The choice of a second-line treatment represents a controversy about the optimal sequence to be proposed: in the VEGFr TKI-resistant setting, is it better to continue treatment with a different VEGFr-TKI or to overcome cross-resistance by switching to a drug with a different mechanism of action? (i.e. a mTORi)? [16-18, 44, 45].

Although switching to a different signalling pathway appears to be rational, current data support the use of both mTORi and TKI: they are associated with similar median progression-free survival (PFS) of about 4-5 months.

However, while axitinib is compared with another TKI in the AXIS trial, everolimus, in the RECORD-1 trial, is compared with placebo.

The AXIS trial is a phase III RCT study comparing axitinib and sorafenib as second line treatment in patients with advanced clear cell RCC, after a prior systemic regimen including sunitinib (54%), cytokines (35%), bevacizumab (8%) or temsirolimus (3%). Although in the overall population, patients treated with axitinib experienced a significantly longer PFS than those on sorafenib (6.7 vs 4.7 months; p<0.0001), in the patients subgroup with disease progression on sunitinib, PFS was shorter with both axitinib and sorafenib (4.8 vs 3.4 months; p<0.0107) than in patients pretreated with cytokines (12.1 vs 6.5 months; p<0.0001) [46].

No OS differences in either axitinib and sorafenib group was detected [47, 48].

As regards mTORi after the VEGFr inhibition-based therapy, data are provided by the RECORD-1 phase trial. In this study patients progressing on first line therapy were randomized to receive everolimus or placebo.

One or two previous VEGFr inhibition based therapies were permitted, including bevacizumab and cytokines. For this reason the results should be interpreted with caution because only 21% of study population were pure second-line therapy progressing after first-line treatment with sunitinib. In this subgroup median PFS was 4.6 with everolimus vs 1.8 months with placebo. 53% of patients received one TKI and cytokine (PFS 5.2 vs 1.8 months) and 26% were third line after two TKIs (PFS 4 vs 1.8 months) [49-52].

Since number and type of previous treatments are crucial, the subsequent RECORD-4 was planned, a phase II trial of only second-line everolimus, which enrolled patients into 3 subgroups based on prior first-line therapy: sunitinib, other anti-VEGF agents (sorafenib, bevacizumab, pazopanib, other) or cytokines. Median PFS was 5.7 months (3.7-11.3) with prior sunitinib, 7.8 months (5.7-11.0) with prior other anti-VEGFs and 12.9 months (2.6-NE) with prior cytokines (preliminary data) [53].

To date there is no direct comparison between the two drugs, axitinib and everolimus, in the post-VEGFr-TKI second-line setting.

Making an indirect comparison between the two mentioned studies in order to identify a drug supported by further evidences, we can summarize that:

- The median PFS with axitinib and everolimus in patients with disease progression after sunitinib in first line setting is similar (4.8 months axitinib vs 4.6 months everolimus); but in the AXIS trial axitinib has an active comparator (sorafenib), while everolimus is compared with placebo;

- In the AXIS trial all population of patients treated with axitinib or sorafenib were pure second-line; in RECORD-1 trial patients were previously treated with one or also two lines of therapy. The final data from RECORD-4, with only second-line everolimus, are still expected.

- Patients randomized to the axitinib arm in the AXIS trial, in absence of adverse events, could have undergone an increase in dose (with potential increased effectiveness), while patients in Everolimus arm in RECORD-1 trial did not undergo this dose escalation.

- No OS difference in AXIS trial was detected. In RECORD-1, patients in treatment with placebo were allowed to cross over to everolimus upon disease progression. Accordingly, OS data are conditioned by the possibility of cross-over.

The first trial that provides a direct comparison between agents with different mechanisms of action is the INTORSECT study; in this trial mRCC patients who had progressed on first-line sunitinib, were randomized to temsirolimus, an mTORi, or sorafenib, a VEGFr TKI.

The difference in median PFS was no statistically significant; median OS was in favor of sorafenib (16.6 vs 12.3 months; *p* = 0.014) [54].

This could suggest an advantage for the VEGFr TKI-VEGFr TKI sequence compared to VEGFr TKI-mTORi sequence, but it really does not clarify the controversy since it compares two treatments that do not represent the best option in the second-line setting.

A study investigating sequential therapies is the RECORD-3 trial. In this phase 2 study, patients were randomized to first line everolimus followed by second-line sunitinib or the opposite sequence, sunitinib followed by everolimus. Results of final analysis do not lead to any change in the standard sequence of sunitinib on first-line followed in second-line by everolimus at disease progression (PFS 21.7 months for everolimus→sunitinib and 22.2 months for sunitinib→everolimus; median OS 22.4 months for everolimus→sunitinib and 29.5 months for sunitinib→everolimus [55, 56].

Also the randomized phase 3 SWITCH-I trial investigating the sequential use of two treatments: sunitinib followed by sorafenib versus sorafenib followed by sunitinib in patients with mRCC without previous treatment, does not change the current guidelines since there was no significant difference in total PFS, first-line PFS, OS and disease control rate between the two arms [57].

Although there are no data about direct comparison between everolimus and axitinib, summarising the available evidence it can be stated that the AXIS trial confirmed the activity of two TKI agents used sequentially (TKI-TKI sequence); RECORD-1 and preliminary RECORD-4 results show the clinical benefit of everolimus in the second line setting but they haven't an active comparator; results of INTORSECT trial that compared a VEGF-TKI with an mTORi as second-line (TKI-mTORi sequence), are not directly transferable in clinical practice.

We can conclude that both everolimus and axitinib are effective options after first-line VEGFr-TKI failure, but the absence of head-to-head comparisons, doesn't solve the controversy for the choice of treatment at present.

Sorafenib might be still considered as an alternative option.

### Treatment beyond second line

Most data related to third-line treatments result from retrospective cohort studies and subgroup analysis: comparative retrospective assessment of the sequence TKI-TKI-mTORi versus TKI-mTORi-TKI suggests superiority of TKI-TKI-mTORi [58]; subgroup analysis within the RECORD-1 trial assessed everolimus as a third-line agent exhibiting a significant benefit regarding PFS versus placebo (4.0 mo PFS vs 1.8 mo; HR: 0.32; p < 0.01), and it is thus in favor of TKI-TKI-mTORi sequence [52].

Treatment in the third-line setting was assessed for the first time in the GOLD trial. In this phase 3 study, patients who received one previous VEGF-TKI inhibitor and one previous mTORi were randomly assigned to receive dovitinib (an oral tyrosine-kinase inhibitor that inhibits VEGFr and FGFr) or sorafenib: PFS difference between sorafenib and dovitinib was not statistically significant (3.6 vs 3.7 months, respectively; HR: 0.86 [0.72-1.04]; *p* = 0.063). Interim OS analysis was also similar in the two arms (11.0 vs 11.1 months, respectively; HR: 0.96 [0.75-1.22]) [59].

This study support the re-treatment with VEGF TKI in third-line, after one previous TKI-mTORi sequence (TKI-mTORi-TKI sequence).

There is also evidence that in patients who have progressed on prior targeted therapy with sunitinib and another TKI or mTORi, the “re-challenge” with sunitinib seems to have a clinical benefit, although with shorter progression-free survival with respect to the first-line treatment [60-63].

These data support the hypothesis that resistance to targeted therapy could be transient.

## OUTLINE OF AN EVIDENCE-BASED THERAPEUTIC ALGORITHM

The optimal sequence of target agent for the treatment of mRCC is not well defined.

**Figure 2:**
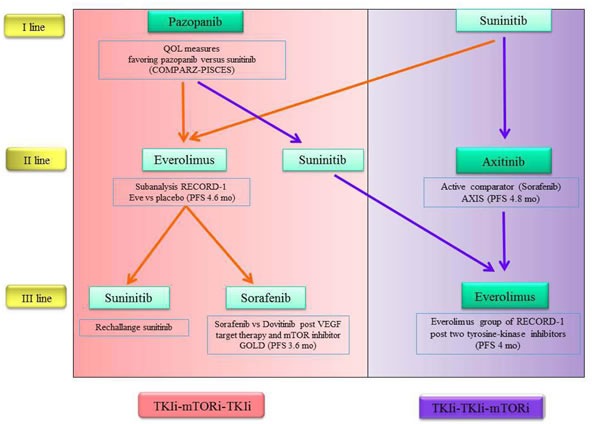
Evidence-based therapeutic algorithm

Assessing the evidence from latest trial, using currently approved oral VEGFR-TKI and mTORi for mRCC, we developed an algorithm, highlighting the strengths of each agents to support his eventual choice (Figure 2).

At the time of the choice of first-line therapy we must primarily take into account primarily the potential efficacy of the drug for the individual patient or the subsequent treatment options?

Given the increased survival of patients with advanced disease and that an increasing number of patient is able to undergoes two or even three lines of therapy, it is necessary a long-term vision and, therefore, planning is better than improvising.

Basing on current drug labels, we can choose between two sequential strategies, which is the best: TKI/TKI/mTORi sequence or TKI/mTORi/TKI sequence?

Unfortunately, to date there are no consistent clinical data to support a clear and strong a sequence rather than another.

Considering the individual drugs, with reference to the first line of treatment, the oral options available today are sunitinib and pazopanib. Both of them have shown equal effectiveness, so it could be use of pazopanib for its better tolerability.

In second line both everolimus and axitinib are associated with similar median PFS: everolimus as a second and third-line therapy show an increase of PFS versus placebo in the soubgroup analysis within RECORD-1 trial.

However the data from AXIS and INTORSECT trials suggested that patients treated with previous TKI may respond to another TKI in second line and cross-resistance may not appear.

To underline that axitinib has an active comparator (sorafenib), as opposed to everolimus (compared with placebo).

Unfortunately it is not possible today to administer today axitinib after pazopanib and this precludes the possibility of using a sequence potentially very effective. Given the similarity between sunitinib and pazopanib, it would be desirable that this may become feasible in the near future.

Treatment for third-line setting depends largely on the choices made previously (Figure 2).

In these TKI-refractory patients it would be appropriate to draw clinical trials with direct comparisons between the drugs, comparing a TKI such as axitinib with a mTORi such as everolimus. This would add important data to choose the best therapeutic sequence.

However, for the decision-making process, should the clinicians consider only evidences arising from individual clinical trials or should they consider also other factors, such as those addressing resistance mechanisms and tumor behavior?

## OUTLINE OF AN ALGORITHM DRIVEN EVEN BY SCIENTIFIC RATIONALE AND TUMOR BEHAVIOR

Because of the challenges still encountered to draw an optimal therapeutic sequence, the integration of evidence-based data with knowledge available about tumor biology could provide complementary information and help clinicians to maximize the effectiveness of targeted agents in the treatment of mRCC.

We have tried to do it starting from the central role of angiogenesis in the pathogenesis of renal tumor. RCC is a hyper-vascular tumor and VEGF have a key role as mediator of angiogenesis in this tumor [64, 65].

Anti-angiogenic drugs typically have transitory efficacy: they produce more or less durable responses, followed by disease progression due to the development of resistance to therapy [37, 38].

From a clinical point of view, indeed, in same patients anti-angiogenic drugs can achieve a good control of the disease for long periods, conversely in other patients there is a very rapid disease progression [66, 67].

We know from literature that resistance to anti-angiogenic treatment is mainly caused by the onset of adaptive mechanisms of cancer cells.

As a response to the inhibition of angiogenesis by VEGF pathway inhibitors, tumor cells promote the transcription of pro-angiogenic factors [68, 69], the recruitment of vascular progenitor cells [70-72] and the increase of the coverage of tumor vessels by pericytes to maintain blood vessels functioning [73-75].

These resistance mechanism may be mediated by the activation of angiogenesis-related pathways independent of VEGFR and PDGFR, which are inhibited by first-line anti-angiogenic drugs [76, 77].

For instance the slow progression under the treatment with sunitinib or pazopanib could be associated with a low capacity to develop adaptive mechanism.

We suppose that in patients with slow progression tumor is still VEGF-dependent.

Therefore we might overcome this transitory resistance by a wider spectrum of receptor inhibition (i.e. sorafenib which blocks VEGFR, PDGFR, Flt-3, c-Kit and Raf-1) or by a stronger inhibition through more potent drugs (i.e. axitinib, which has a stronger inhibitory potency in terms of IC50 [78, 79]).

Since AXIS trial provided strong data about the superiority of axitinib over sorafenib in this setting of patients, axitinib should be the best option as second-line treatment for patients experiencing slow progression during firs-line sunitinib or pazopanib.

The patients who received a TKI and subsequently a different TKI, could undergo a third-line treatment with a further TKI or the mTORi everolimus.

However the use of a third-line TKI (i.e. sunitinib or sorafenib) after two previous lines of TKIs is not supported by strong evidence-based data. Conversely the use of everolimus after TKIs in first and second-line treatment met favorable outcomes in RECORD-1 trial.

Patients who develop a rapid progression during firs-line TKI may have an intrinsic primary resistance to anti-angiogenic drugs, so that the resistance mechanism would not be adaptive. Thus in these patients we should address a different pathway.

mTOR inhibition by everolimus could be the most valid option as second-line treatment.

**Figure 3:**
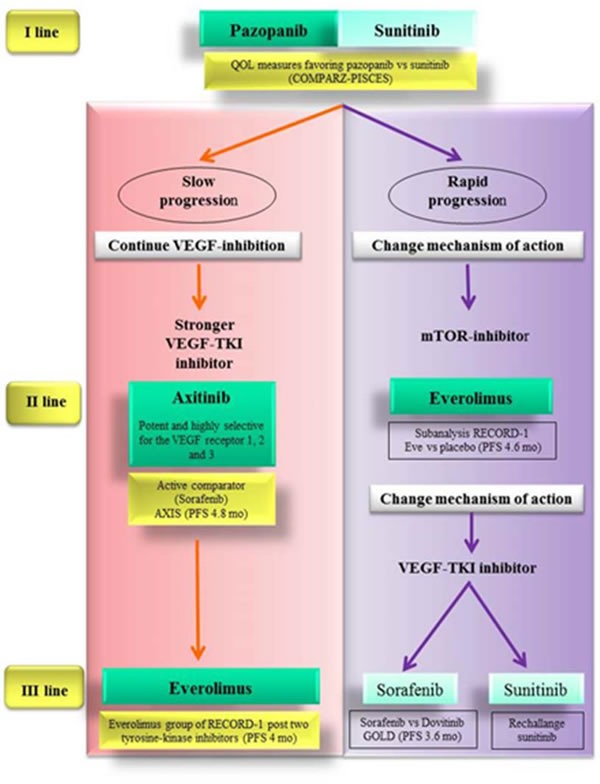
Evidence-based therapeutic algorithm driven also by scientific rationale and tumor behavior

Accordingly for third-line treatment in this subset of patients a change of mechanism of action appears more reasonable. So after first-line TKI and second-line mTORi, a different TKI could be chosen as third-line treatment. Sunitinib is supported by retrospective studies about rechallange. As an alternative option sorafenib is sustained by data from the GOLD study (Figure 3).

## EMERGING TREATMENT

Ongoing trials and other recently concluded [80-83] continue to research new therapies and to elucidate the optimal sequence of the known ones.

Recently, Immunoncology, represent a new and promising frontier for many malignancies, such as RCC.

The aim of Immunotherapy is to improve the organism's competence to direct the immune system against cancer cells.

The normal immune system requires T cell activation for its activity; some immunomodulatory molecules such as cytotoxic T-lymphocyte associated antigen-4 (CTLA-4) and programmed death-1 (PD-1), are involved in the mechanism for T cells activation [84, 85].

They are inhibitory molecules, dysregulated in several cancer, resulting in defective ability of immune system to react against tumor cells.

As a consequence, checkpoint inhibitors have been developed and are under evaluation [86-92].

Nivolumab, a fully human IgG4 monoconal antibody directed against PD1, demonstrated effectiveness in patients with metastatic melanoma and non-small cell lung cancer.

In a phase II trial three nivolumab doses (0.3, 2 or 10 mg/kg IV Q3W) have been assessed in mRCC patients pretreated with VEGFR TKIs, mTORi and immunotherapy. No dose-response relationship was detected for PFS; median OS was 18.2 months by the 0.3 mg/kg dose and was not reached by other doses [93].

Basing on data from CheckMate 016 trial, a phase I study of nivolumab + ipilimumab (a human CTLA-4-blocking antibody) in patients with mRCC [94, 95], a phase III trial was designed to evaluate nivolumab + ipilimumab compared with sunitinib monotherapy for previously untreated mRCC has recently been planned [96].

Recently the results of two pivotal, randomized, phase 3 trial, about nivolumab and cabozantinib, respectively, compared with everolimus have been published: both provide substantial benefit in patients treated with previous VEGF targeted therapy.

The first, CheckMate 025, an open-label phase 3 trial, investigates nivolumab versus everolimus in patients who have failed one or more anti-VEGF therapy.

The median PFS was similar (4.6 months with nivolumab and 4.4 months with everolimus) while Nivolumab showed considerable OS advantage and tolerability over everolimus (OS 25.0 months with nivolumab and 19.6 months with everolimus) [97].

The second randomised phase 3 trial (METEOR) compared everolimus with cabozantinib, a small-molecule tyrosine kinase inhibitor of MET, VEGFR2 and AXL. The setting tested was the second-line after failure of one line or more of VEGF targeted therapy.

Cabozantinib showed superior PFS (7.4 mo) versus everolimus (3.8 mo). Grade 3 or 4 adverse events were more numerous in cabozantinib sub-group (68%) than everolimus-group (58%).

In the interim analysis is evident a trend of cabozantinib toward an OS advantage but the final planned mature OS data is expected in 2016 [81].

**Figure 4:**
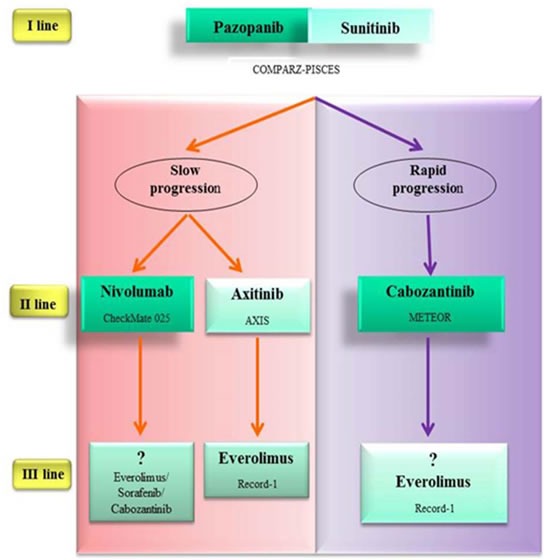
Promising therapeutic algorithm in the near future Nivolumab and cabozantinib are superior to everolimus in patients who have failed one line or more of VEGF targeted therapy (CheckMate 025 study; METEOR study). Nivolumab showed delayed benefit in PFS versus everolimus. The progression-free survival curves has a late separation in the study. Therefore it would seem reasonable to use it in fit patients with slow progression. Cabozantinib is a multi-tyrosine kinase inhibitor. For its impressive PFS versus everolimus (cabozantinib median PFS 7.4 mo; everolimus median PFS 3.8 mo) should be considered for patients with rapid progression. No trials have compared these two experimental agents directly against axitinib in the second-line setting, where axitinib showed no OS advantage. Survival advantage and tolerability profile of nivolumab over everolimus makes it a valid option also versus axitinib.

Both these new promising drugs, nivolumab and cabozantinib, not yet be used in the clinical practice. With their next introduction, a promising therapeutic algorithm will be available in the near future (Figure 4).

## CONCLUSIONS

Physicians now have many more tools to improve outcomes of mRCC.

Planning the appropriate and personalized sequence of targeted agents may help to achieve the best clinical outcome in these patients by the maximum number of available targeted agents.

Given the short differences in median PFS observed between treatments, in the absence of clinical trials with direct comparisons between the drugs, the optimal therapeutic sequence is still under investigation.

Our opinion is that, in addition to evidences from clinical trials, other factors must be also considered for decision making such as the safety profile of drugs [98, 99], comorbidities of the patient and tumor behavior.

To integrate evidence-based data from trials with clinical observations about tumor biology could provide complementary information and help clinicians to individualize the treatment and to generate the optimal sequence for each patient.

New and promising agents, such as immune based therapies, continue to be explored; data from ongoing trial will help to develop more effective therapeutic strategies and new algorithms.
